# Abernethy as a Lecturer

**Published:** 1840-06

**Authors:** 


*Abernethy as a Lecturer.*—The lecture-room was the grand theatre upon which Mr. Abernethy displayed; there, indeed, he "shone eccentric like a comet's blaze!" and there he would indulge his disposition and propensities to an extent which occasioned the pupils frequently to regard it as an exhibition, and call it an "Abernethy at Home." His mode of entering the lecture-room was often irresistibly droll—his hands buried deep in his breeches-pockets, his body bent slouchingly forward, blowing or whistling, his eyes twinkling beneath their arches, and his lower jaw thrown considerably beneath the upper. Then he would cast himself into a chair, swing one of his legs over an arm of it, and commence his lecture in the most *outre* manner. The abruptness, however, never failed to command silence, and rivet attention.

"'The Count was wounded in the arm—the bullet had sunk into the flesh—it was, however, extracted—and he is now in a fair way of recovery.' That will do very well for a novel, but it won't do for us Gentlemen: for 'Sir Ralph Abercromby received a ball in the thick part of his thigh, and it buried itself deep: and it got among important parts, and it couldn't be felt; but the surgeons, nothing daunted, groped, and groped, and groped,———and Sir Ralph died.'"

